# Intra‐individual changes in sperm parameters and total motile count with time among infertile men

**DOI:** 10.1111/andr.13638

**Published:** 2024-04-30

**Authors:** Gilad Karavani, Bader Akroof, Susan Lau, Kirk C. Lo, Ethan D. Grober, Vrati M. Mehra, Mohamed S. Kattan, Katherine Lajkosz, Keith Jarvi

**Affiliations:** ^1^ Department of Surgery Division of Urology Mount Sinai Hospital University of Toronto Toronto Canada; ^2^ Department of Surgery Faculty of Medicine University of Toronto Toronto Canada; ^3^ Department of Biostatistics Princess Margaret Cancer Centre Toronto Canada

**Keywords:** intra‐uterine insemination, in‐vitro fertilization, male infertility, spermatozoa, total motile count

## Abstract

**Background:**

Paternal age association with sperm parameters has been previously studied, demonstrating a decrease in semen volume, sperm motility, and sperm morphology, but not in sperm concentration. However, scarce data exists on the individual intra‐personal changes in semen parameters with time.

**Study design:**

Retrospective cohort study.

**Objective:**

To evaluate the changes in semen parameters and total motile count of infertile men over time.

**Materials and methods:**

In this retrospective cohort study, infertile men without known risk factors for sperm quality deterioration and at least two semen analyses done > 3 months apart, between 2005 and 2021, were evaluated. Allocation to groups was according to time between first and last semen analyses – 3–12 months, 1–3 years, 3–5 years, and > 5 years. Basic characteristics and first and last semen analyses were compared. The primary outcome was the change in sperm parameters and the secondary outcome was the occurrence of a total motile count < 5 million in men with an initial total motile count > 10 million.

**Results:**

A total of 2018 men were included in the study. The median age at first semen analyses was 36.2 (interquartile range: 32.8–40.1) years and the median time between semen analyses was 323 days (range 90–5810 days). The overall trend demonstrated an increase in concentration in the 3–12 months and the 1–3 years groups, whereas volume, motility, and morphology remained similar in these time groups. Semen analyses done more than 5 years apart showed decreased volume (*p* < 0.05), motility (*p* < 0.05) morphology (*p* < 0.05), and steady sperm concentration. Significant declines in TMCs were found over time (*p* < 0.001), with 18% and 22% of infertile men with an initial total motile count > 10 million dropping to < 5 million after 3 and 5 years, respectively. The factors independently predictive of total motile count < 5 M in the last semen analyses in men with an initial total motile count of > 10 M in a multivariate logistic regression model were baseline volume (odds ratio 0.80, *p* = 0.03), baseline total motile count (odds ratio 0.98, *p* = 0.01) and time between semen analyses – 3–5 years (odds ratio 3.79, *p* < 0.001) and > 5 years (odds ratio 3.49, *p* = 0.04)

**Discussion:**

Our study demonstrates, at the individual level, that while improvement in sperm concentration is observed in the first year and between 1 and 3 years, possibly due to fertility treatments, fertility‐related counseling, and lifestyle changes, semen parameters decline with time over 3 years in individuals. Of significance, close to 22% of men with an initial total motile count > 10 million (a range where spontaneous pregnancy is attainable) declined to < 5 million (a range usually indicating a need for in‐vitro fertilization/intracytoplasmic sperm injection) over 5 years. This data could contribute to individualized family planning for infertile men regarding the mode and timing of conception and the need for sperm banking, in order to minimize the need for future fertility treatments.

## 1 INTRODUCTION

Male factor infertility accounts for half of the infertility‐related difficulties faced by couples.^1^ Globally, approximately 1 male in every 10 suffers from infertility or subfertility.^2^ Some of these men have genetic conditions that lead to reduced sperm counts (oligospermia) or no spermatozoa at all (azoospermia), while others may develop oligospermia or experience secondary infertility over time. Many factors, in addition to genetic conditions, have been shown to impact semen quality parameters such as sperm concentration and motility. Some of these include endocrine‐disrupting chemicals including phthalates, pesticides, and herbicides,^3^ smoking,^4^ and BMI,^5^ year of birth,^6^ geographical location,^7^ and paternal age.^8^

Ever since the publication of the Danish study led by Carlsen et al. in 1992, which reported a 50% decrease in sperm production from 1940 to 1990 (*p* < 0.0001), research on semen quality has drawn global attention.^9^ This has led to the publication of several studies and meta‐analyses examining semen quality over time. Most of these have focused on exploring changes in sperm parameters in men from different generational cohorts to assess changes in semen parameters over long periods of time.^10^ While this type of analysis is insightful, the model may present several limitations such as the inability to account for changes in laboratory testing between different labs, changes in technology over time, as well as patient‐specific factors such as health status, fertility status, and ethnicity. It also does not allow us to understand how semen parameters change with time at the individual level. Having said that, a handful of studies have evaluated and tracked the changes in the semen quality of a sample of men longitudinally.^11^ However, these have some limitations including a narrow age range of participants not allowing longitudinal assessment of changes in semen with aging, small sample sizes, inability to account for changes in semen volume or morphology, and/or brief follow‐up time.^11^ There is a need for larger‐scale studies examining intra‐individual changes in sperm quality, count, and concentration, longitudinally in men over clinically significant periods of time. Doing so would enable clinicians to better guide patients on family planning with regard to the mode and timing of conception in order to minimize the need for future fertility treatments.

As such, our study aimed to track and assess changes in semen parameters and total motile count (TMC) among a large sample of infertile men with at least two semen analyses done over short and long time intervals.

## 2 METHODS

### 2.1 Study population

This retrospective cohort included data from a single Canadian referral institution which was prospectively collected. We retrospectively reviewed data from our study population—infertile men who attended our male infertility clinic for evaluation between 2005 and 2020 and completed an intake questionnaire upon the first visit. This questionnaire included demographic data, lifestyle‐related data including substances used, and prior medical and reproductive background. Medication use included treatment for diabetes, hypertension, depression/anxiety, hypo/hyperthyroidism, anti‐inflammatory drugs, 5‐aminosalicylic acid, corticosteroids, statins, aspirin, and anti‐coagulants. Men included in this study had at least two semen analyses, done 3 months or more apart, at our spermatozoa laboratory. Men who delivered their second semen analysis (SA) at a different laboratory were not included in this study. Exclusion criteria were exposure to risk factors known to affect semen quality—chemotherapy, radiotherapy, testicular or other cancer diagnosis, past vasectomy, and cases of Klinefelter syndrome diagnosis.

Men who had azoospermia in their baseline SA or had only a single SA were also excluded from the study.

### 2.2 Outcomes

The primary outcome of this study was the change in sperm parameters between the baseline SA and last SA and the secondary outcome was the occurrence of low TMC—under 5 million with time in those who had an initial TMC of more than 10 million.

The study population was divided into four groups according to the time interval between their baseline SA and their last documented SA—under 1 year, 1−3 years, 3−5 years, and over 5 years. We compared basic characteristics, reproductive data, and baseline and last sperm parameters between the 4 groups. We evaluated the mean change per patient in each sperm parameter in all four groups. Additionally, a subgroup analysis by age groups (under 40, 40−50, and above 50 years) was performed, comparing intra‐individual differences in sperm parameters between the time groups. Finally, we created univariate and multivariate logistic regression models for the prediction of TMC drop from above 10 million to under 5 million between semen analyses in the entire population.

### 2.3 Specimen collection and evaluation

Semen samples were collected after 2−7 days of abstinence and provided by means of masturbation. All samples were evaluated in the same Andrology laboratory, at Mount Sinai Hospital, Toronto, Canada. Semen was analyzed according to 2010 WHO criteria^12^ for volume (milliliter (mL)), concentration (million/mL), motility (%), progressive motility (%), normal morphology (%), viability (%), and TMC—total number of motile spermatozoa in the entire sample (million).

Two specimens from each semen sample were analyzed to determine the value of sperm concentration and motility by Mackler chamber combined with computer‐assisted sperm analysis and sperm morphology was assessed by microscopic evaluation of spermatozoa for structural characteristics. Standardized SOPs following the WHO Manual guidelines were used in the Andrology Laboratory for the analysis of the semen samples, which did not materially change during the course of the study. Quality control for consistency was performed on an ongoing and regular basis in the Andrology laboratory.

The interpretation of the SA results (to interpret as normal or outside of the normal limits) changed with the changes in the WHO Manuals.

### 2.4 Statistical analysis

Categorical characteristics were summarized as frequencies and proportions and differences across time interval groups were assessed using the Chi‐squared test or Fisher's exact test, as indicated. Continuous characteristics were summarized using means and standard deviations, and differences were assessed using the Kruskal‐Wallis test. For sperm parameters that were statistically significant (*p* < 0.05) in the omnibus test, post‐hoc pairwise tests with the Holm correction were conducted to identify pairwise differences. To assess if differences in parameter change remain after adjusting for baseline values and age, multivariable linear regression models using the time between SA, baseline parameter values, and baseline age to predict final parameter values were fit.

Additionally, a logistic regression model was applied to search for independent predictors for the dichotomous dependent parameter of the last TMC of less than 5 million in patients with an initial TMC of 10 million or more, reporting odds ratio (OR) and 95% confidence interval (CI) for each parameter. This was done using a univariate and multivariate logistic regression model. A *p*‐value of < 0.05 was considered statistically significant, with all tests being two‐tailed. We used the 4.0.0 version of the R Foundation for Statistical Computing for the statistical analysis.

### 2.5 Ethical approval

The Research Ethics Board approved data collection and analysis (reference numbers 05‐0161‐E and 07‐0032‐E, respectively) The date of the approval was October 18, 2005, for data collection and October 30, 2007, for data analysis. An Institutional Review Board‐approved informed consent form was signed by all participants prior to questionnaire completion and the principles of the Helsinki Declaration (2013) were followed.

## 3 RESULTS

Overall, 6854 men were evaluated in our male infertility clinic and had a semen analysis performed at our center. We excluded 1943 men with azoospermia or background of previous exposure to chemotherapy or radiotherapy, testicular or other cancers, past vasectomy, and men with Klinefelter syndrome. Of the remaining 4911 men, 2018 had at least one more SA done at the same laboratory and at a minimal interval of 3 months between the semen analyses and were included in the final analysis.

The median age at baseline SA was 36.2 (interquartile range [IQR]: 32.8–40.1) years and the median time between tests was 323 days (range 90–5810 days). Basic characteristics and sperm parameters information were compared between the 4 time groups (**Table** **1**)—less than 1 year, 1−3 years, 3−5 years, and more than 5 years. Differences were noted between groups in age at baseline SA, diabetes, duration of infertility, use of medication, and mean sperm concentration at baseline SA.

**TABLE 1:** Characteristics of the study population at first visit to the infertility clinic—According to the time between the baseline and last semen test.

	Time between first and last semen analysis				
Parameter	1 year>	1–3 years	3–5 years	> 5 years	*p*‐value
**Number of patients**	1179	594	154	91	
**Age (years)**	36.6 (33.0, 40.7)	36.0 (32.9, 39.5)	36.4 (32.3, 40.8)	33.6 (30.7, 37.1)	<0.001
**BMI**	27.4 (25.1, 30.5)	27.1 (24.4, 30.3)	27.2 (24.9, 29.1)	26.6 (23.9, 28.4)	0.37
**Smoker** ^*^	87/1179 (7.4%)	56/594 (9.4%)	8/154 (5.2%)	8/91 (8.8%)	0.26
**Alcohol use** ^*^	647/1179 (54.9%)	307/594 (51.7%)	76/154 (49.4%)	47/91 (51.6%)	0.41
**Marijuana use** ^*^	122/1179 (10.3%)	65/594 (10.9%)	20/154 (13.0%)	12/91 (13.2%)	0.67
**Diabetes mellitus** ^*^	35/1179 (3.0%)	38/594 (6.4%)	7/154 (4.5%)	7/91 (7.7%)	0.003
**Hypertension**	31/1169 (2.6%)	13/594 (2.2%)	4/154 (2.6%)	2/91 (2.2%)	0.96
**Years of infertility**	2.00 (1.20, 3.50)	2.00 (1.00, 3.00)	2.70 (1.50, 5.00)	2.00 (1.00, 5.00)	0.04
**Primary infertility** ^*^	747/871 (85.8%)	436/494 (88.3%)	127/142 (89.4%)	67/80 (83.08)	0.36
**Medications use** ^*^	282/1179 (23.9%)	180/594 (30.3%)	46/154 (29.9%)	29/91 (31.9%)	0.01
**First semen analysis**					
**Volume (mL)**	2.50 (2.00, 3.50)	2.50 (2.00, 4.00)	2.50 (1.50, 4.00)	3.00 (2.00, 4.00)	0.9
**Concentration (M/mL)**	12.9 (3.70, 35.0)	11.3 (2.90, 32.7)	17.0 (3.50, 45.6)	8.70 (0.90, 29.3)	0.03
**Motility (%)**	21.1 (12.7, 28.7)	22.2 (12.7, 32.4)	22.2 (14.0, 33.2)	20.0 (9.40, 31.1)	0.3
**Normal morphology (%)**	10.0 (5.00, 15.0)	10.0 (5.00, 15.0)	10.0 (5.00, 20.0)	10.0 (5.00, 20.0)	0.05
**Viability (%)**	70.0 (55.0, 75.0)	70.0 (60.0, 75.0)	70.0 (50.0, 77.5)	65.0 (55.0, 75.0)	0.07
**Total motile count (millions)**	5.80 (1.24, 20.3)	5.43 (1.08, 19.5)	7.36 (1.02, 35.2)	3.30 (0.37, 19.3)	0.18

*Note*: Data are presented as *n*/*N* (%) or median (Q1, Q3). *p*‐Values for continuous and categorical characteristics are from are from the Kruskall‐Wallis and Fisher Exact tests, respectively.

Abbreviations: BMI, body mass index.

* This information was obtained from patient‐completed standardized online questionnaires. The information obtained was smoker (Yes, No) and if yes, the number of packs/day, alcohol use (Yes, No) and if yes, the amount consumed daily, Marijuana use (Yes, No), diabetes mellitus (Yes, No), medication use (Yes, No) and if yes, then the patient was asked to list the types of medications; primary infertility was based upon previously established pregnancies.

Intrapersonal changes in semen parameters between SA were calculated and the average difference per patient was evaluated for each time period (Table 2). The overall trend was improved concentration in the first year and the 1−3 years period, whereas semen volume, sperm morphology, and motility remained similar in these time intervals. Nevertheless, semen analyses done more than 5 years apart demonstrated a shift toward a decrease in sperm quality—showing lower volume, concentration, motility, and morphology in the longer time interval between SA.

**TABLE 2:** Changes in sperm parameters by time between baseline and last semen analyses.

	Time between baseline and last semen analysis				
	<1 year	1–3 years	3–5 years	>5 years	*p*‐value
**No. of patients**	1179	594	154	91	
**Age at last SA (years)**	37.0 (33.4, 41.2)	37.6 (34.4, 41.0)	40.0 (36.4, 44.5)	40.8 (37.5, 44.2)	**<0.001**
**Last volume (mL)**	2.50 (2.00, 3.50)	2.50 (1.80, 3.50)	2.50 (1.50, 3.50)	2.50 (1.50, 3.00)	**0.04**
**Change in volume**	0.00 (−0.50, 0.50)	0.00 (−0.50, 0.50)	−0.15 (−1.00, 0.50)	−0.10 (−1.00, 0.42)*	0.08
**Last Concentration (million/mL)**	18.1 (4.45, 43.5)	17.1 (4.50, 44.1)	17.2 (3.32, 43.2)	7.20 (0.50, 27.7)	**<0.001**
**Change in concentration**	0.80 (−3.85, 10.5)	0.80 (−3.00, 10.7)	0.00 (−7.65, 10.1)	−0.10 (−9.45, 4.08)	0.29
**Last motility (%)**	22.3 (12.8, 31.6)	23.0 (14.3, 32.5)	18.8 (12.9, 30.9)	15.6 (4.40, 26.2)	**0.002**
**Change in motility**	0.50 (−4.20, 6.33)	0.85 (−4.52, 6.43)	−1.40 (−9.00, 6.10)*	−1.30 (−9.07, 2.40)*	**<0.001**
**Last morphology (%)**	10.0 (5.00, 15.0)	10.0 (5.00, 15.0)	10.0 (5.00, 15.0)	5.00 (2.00, 15.0)	0.07
**Change in morphology**	0.00 (−1.00, 3.00)	0.00 (−1.00, 3.00)	0.00 (−5.00, 0.00)*	−1.00 (−8.00, 0.00)*	**<0.001**
**Last viability (%)**	70.0 (55.0, 75.0)	70.0 (55.0, 75.0)	60.0 (45.0, 75.0)	60.0 (43.8, 75.0)	**0.002**
**Change in viability**	0.00 (−5.00, 10.0)	0.00 (−10.0, 5.00)	0.00 (−10.0, 5.00)	−5.00 (−10.0, 5.00)	0.18
**Last total motile count (millions)**	7.93 (1.67, 27.1)	8.08 (1.80, 27.1)	8.03 (0.93, 29.0)	1.69 (0.28, 15.1)	**<0.001**
**Change in total motile count (millions)**	0.33 (−1.89, 8.27)	0.42 (−2.81, 7.47)	−0.12 (−9.18, 4.79)	−0.70 (−6.03, 0.99)	**<0.001**

Data are presented as median (Q1, Q3).

*Note*: SA, semen analysis *p < 0.05 for the comparison between this group and the < 1 year group.

**FIGURE 1:**
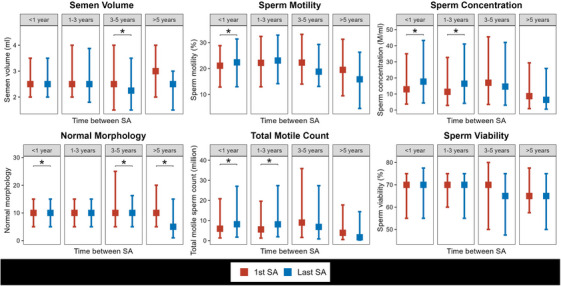
Median (IQR) sperm parameters at baseline and final assessment by time interval for each group of patients. Results are stratified by timing between baseline and final assessments. *Note*: SA, semen analysis. *p < 0.05 from Wilcoxon signed‐rank test compares change between the baseline and last semen analysis.

We compared the average intra‐personal level of change between the 4 time groups. This analysis showed that the median [IQR] change in semen volume differed between < 1 year and the > 5 years groups (0.00 [−0.50, 0.50] vs. −0.10 [−1.00, 0.42], *p* = 0.03). This was also the case for the change in sperm motility and morphology, with a significant difference between the < 1 year, the 3−5 years, and > 5 years groups (Table 2). The results are held in the sensitivity analysis (Table S1). Results are visualized in Figure 1.

An additional subgroup analysis for sperm parameters changes by age groups at baseline SA—under 40 years (*n* = 1491), 40−50 years (*n* = 455), and above 50 years (*n* = 72)—was performed. In the younger than 40 years subgroup, a significant difference was noted over time in all variables (except for semen volume)—motility, concentration, morphology, and TMC with a similar trend of improvement in the first 3 years and a gradual decrease in the 3−5 years and > 5 years intervals. However, in the 40−50 years and older than 50 years groups, semen parameters changes were similar between time groups for nearly all parameters (except for morphology in the 40−50 years group (which improved in the first 3 years and then decreased) and motility in the > 50 years group (decrease in the first 3 years and improvement in the 3−5 years) (Table S2).

The secondary outcome investigated—the occurrence of TMC drop to below 5 million in men who initially had a TMC above 10 million—was tested and further investigated using logistic regression models predicting TMC < 5 at the last assessment. Of the 719 patients with a baseline TMC of 10 million or more, 61 ultimately had TMC < 5 at last SA. A significant decline in TMCs to below 5 million was found in the 3−5 years and > 5 years group, with 18% and 22% of the men with an initial TMC > 10 million, compared to 7.2% and 7.1% in the 1−3 years and < 1‐year groups, respectively (*p* < 0.05 all comparisons).

We additionally performed univariate and multivariate logistic regression models for the prediction of TMC < 5 M in those with a baseline TMC > 10 M. The multivariate model (Table 3) adjusted for predictors that were significant in the univariate analysis ‐baseline semen volume, sperm motility, and morphology, TMC, and time between SA (categorized). This regression model demonstrated that the factors independently predictive of TMC dropping below 5 million in the last SA were baseline semen volume (odds ratio [OR] 0.80 [95% confidence interval {CI}: 0.65–0.98], *p* = 0.03), baseline TMC (OR 0.98 [95% CI 0.96–0.99], *p* = 0.01) and time between SA – 3−5 years (OR 3.79 [95% CI 1.72–8.38], *p *< 0.001) and > 5 years (OR 3.49 [95% CI 1.05–11.59], *p *= 0.04)

**TABLE 3:** Multivariate logistic regression models for prediction of total motile sperm count drop from above 10 million to under 5 million.

Parameter	OR	95% CI	*p*‐value
**Baseline semen volume (mL)**	0.80	0.65–0.98	**0.03**
**Baseline sperm motility (%)**	0.99	0.96–1.02	0.57
**Baseline total motile count (millions)**	0.98	0.96–0.99	**0.005**
**Baseline sperm morphology (%)**	0.98	0.95–1.02	0.30
**Time between semen analyses**			
** < 1 year**	Reference		
**1–3 years**	1.17	0.60–2.30	0.64
**3–5 years**	3.79	1.72–8.38	**<0.001**
** > 5 years**	3.49	1.05–11.59	**0.04**

## 4 DISCUSSION

In this study, we explored the intra‐individual changes in sperm parameters and TMC over time in infertile men, in order to identify and predict deterioration in sperm quality in different time periods since the first SA. We were able to demonstrate that sperm parameters either improve or remain similar in the first 5 years. More specifically, within the first year, sperm concentration, motility, and morphology are improved in a repeat SA. This improvement then subsides in the 1−5‐year period (except for sperm concentration, which remains higher within the 1−3‐year period and unchanged in the 3−5‐year period post baseline SA). However, in an observation of SA done 5 years or more apart, a significant drop in semen volume, sperm motility, and sperm morphology are noted, while concentration and TMC remain unchanged. Moreover, the multivariate regression model for prediction of TMC decrease to below the clinically relevant cut‐off value of 5 million shows that lower volume, lower initial TMC, and time periods of over 3 and 5 years since baseline SA are predictive of subsequent occurrence of TMC under 5 million.

It is noteworthy that high intra‐individual variability and significant fluctuation in semen analysis results may be found over time, as seen in healthy volunteers who provided semen analyses for a placebo arm.^12^ Consequently, when interpreting our findings, it is crucial to take into account the potential overlap between fertile and subfertile men. Many factors have been associated with declining spermatogenesis, including age, environmental exposure, and chronic inflammation and illness.^13^, ^14^, ^15^, ^16^, ^17^, ^18^ Several meta‐analyses investigating the effect of advanced paternal age on sperm parameters have shown conflicting results regarding the decrease in sperm quality with age. However, though debatable, most studies did show a gradual age‐related decrease in volume, motility, and morphology, and a relatively unchanged sperm concentration.^19^, ^20^, ^21^, ^22^

These studies are in accordance with our results of similar sperm parameters in the first five years and the later worsening in sperm parameters, except for sperm concentration. The time intervals reflect, at the individual level, the age advancement effect and accumulation of harmful exogenic and endogenic exposures, gradually affecting sperm quality. We speculate that the short‐term (first year) improvement in most sperm parameters is likely in response to fertility therapies including lifestyle modifications (cessation of smoking, alcohol, cannabis consumption, healthy diet, and physical activity), initiation of fertility supplements intake (that contain Coenzyme Q10, Acetyl L‐Carnitine, and vitamins B12, C, and E) and possible medical (antibiotics and/or anti‐inflammatory drugs for suspected prostatitis, urinary tract infection, epididymitis or other inflammatory conditions of the genito‐urinary tract) or surgical interventions (such as varicocelectomy, bariatric surgery, etc.), while the long‐term worsening in all but sperm concentration may represent the natural course of sperm quality evolution in men with time, which reflects longer exposure to environmental factors (increased oxidative stress) and the occurrence of acute or chronic medical conditions, as well as weight gain and drug use, over the years. TMC is an important predictive parameter for spontaneous conception, intra‐uterine insemination (IUI) outcomes, and assisted reproductive technology–in‐vitro fertilization (ART–IVF) outcomes and is used as a means to determine which couples might benefit most from the type of ART offered (IUI, IVF, or intracytoplasmic sperm injection [ICSI]).^23^, ^24^, ^25^ TMC under 10 million was previously shown to result in a decreased chance for spontaneous pregnancies and poorer IUI outcomes.^26^, ^27^ Moreover, TMC < 5 million spermatozoa indicated the need for an IVF cycle due to very low IUI success rates, and ICSI use is indicated for those with TMC < 1 million spermatozoa.^28^, ^29^ Our regression model shows that even men who currently have a TMC > 10 million are at risk for a future decrease to below 5 million after 3 years, even after adjustment for their initial TMC and semen volume.

The significance of the male infertility workup, diagnosis, and treatment cannot be overstated. While the primary objective of addressing male infertility is to achieve pregnancy and parenthood, it is crucial to underscore that research indicates a link between infertility and later ramifications such as anxiety and depression.^30^, ^31^ Consequently, successful fertility treatments not only aim for reproductive success but also carry the additional benefit of positively impacting mental health. Furthermore, recent studies suggest that semen quality may serve as a potential biomarker for future general health. This association extends to a higher incidence of chronic conditions, including autoimmune diseases, diabetes, cardiovascular disease, hypertension, and an elevated risk for various cancers such as testicular, prostate, thyroid, melanoma, and hematological malignancies. Thus, the evaluation and diagnosis of male infertility present an opportunity to enhance current overall health and underscore the importance of future screening and prevention of significant future general health conditions.^32^, ^33^

This study has several limitations, the first and main being its retrospective nature. Another limitation derived from the retrospective design is the lack of data on time of abstinence, laboratory evaluation, lifestyle, behavior and nutrition alteration, and medical and surgical interventions done including a diagnosis of varicocele and its repair, all of whom might affect sperm parameters and the natural course of changes in its quality. Nevertheless, most of these modifications' effects would probably be expressed in the first year or two and have a mild, if any, effect on the remaining time intervals.

This study provides a unique overview, at the individual level, of changes over time according to the initial semen analysis of infertile men and is of importance for counseling patients regarding future fertility potential and possible challenges based on expected time course and current sperm parameters.

To conclude, Intra‐individual variation in sperm parameters follows a trend of short‐term improvement, followed by a revert to baseline in the 1−5 years and a later decline in all parameters except for sperm concentration and TMC. The longer time interval of over 3 years from the first SA, as well as lower initial TMC, is predictive of TMC drop under 5 million. There is a need for prospective, longitudinal studies examining intra‐individual changes in sperm quality, count, and concentration in infertile men over clinically significant periods of time in order to enable broader applicability of our results. Doing so would enable clinicians to better guide patients regarding family planning in order to minimize the need for future fertility‐related interventions.

## AUTHOR CONTRIBUTIONS

*Conceptualization*: Gilad Karavani, Katherine Lajkosz, and Keith Jarvi. *Data curation*: Gilad Karavani, Bader Akroof, Mohamed S. Kattan, Kirk C. Lo, Ethan D. Grober, and Vrati M. Mehra. *Formal analysis*: Katherine Lajkosz. *Investigation*: Keith Jarvi and Gilad Karavani. *Methodology*: Gilad Karavani, Katherine Lajkosz, Susan Lau, Kirk C. Lo, Ethan D. Grober, and Keith Jarvi. *Project administration*: Susan Lau. Software: Katherine Lajkosz. *Supervision*: Keith Jarvi. *Validation*: Katherine Lajkosz and Gilad Karavani. *Writing—original draft*: Gilad Karavani, Vrati M. Mehra, Katherine Lajkosz, and Keith Jarvi. *Writing—review & editing*: Gilad Karavani, Ethan D. Grober, Kirk C. Lo, Bader Akroof, Mohamed S. Kattan, Bader Akroof, Susan Lau, and Keith Jarvi. *Final approval*: all authors.

## CONFLICT OF INTEREST STATEMENT

The authors declare no conflict of interest.

## FUNDING INFORMATION

This study did not receive funds.

## Supporting information

Supporting Information

Supporting Information

## Data Availability

The data that support the findings of this study are available on request from the corresponding author. The data are not publicly available due to privacy or ethical restrictions.
