# Supplementary material for: Intra‐individual changes in sperm parameters and total motile count with time among infertile men
Source: Andrology. 2024 Apr 30;13(2):226–33. doi: 10.1111/andr.13638 (PMC11815539; doi:10.1111/andr.13638)
Supplement: Supplementary file 1 — Supporting Information [file ANDR-13-226-s002.docx]

**Suppl. Table 1**. Multivariable regression models predicting final sperm parameter values by time between sperm assessments, adjusting for baseline value and age.

| **Sperm Parameter** | **Parameter** | | **Estimate (95% CI)** | **P-Value** |
| --- | --- | --- | --- | --- |
| **Volume** | Baseline volume |  | 0.66 (0.63, 0.70) | <0.001 |
|  | Baseline age |  | -0.01 (-0.02, -0.005) | **0.002** |
|  | Time between SA | 3 months – 1 year | Reference |  |
|  |  | 1-3 years | 0.004 (-0.12, 0.11) | 0.95 |
|  |  | 3-5 years | -0.18 (-0.38, 0.02) | 0.08 |
|  |  | >5 years | -0.33 (-0.57, -0.09) | **0.008** |
| **Motility** | Sperm motility |  | 0.70 (0.66, 0.73) | <0.001 |
|  | Baseline age |  | -0.15 (-0.23, -0.07) | <0.001 |
|  | Time between SA | 3 months – 1 year | Reference |  |
|  |  | 1-3 years | -0.03 (-1.09, 1.03) | 0.95 |
|  |  | 3-5 years | -2.30 (-4.18, -0.41) | 0.02 |
|  |  | >5 years | -5.24 (-7.68, -2.79) | <0.001 |
| **Sperm count** | Baseline sperm count |  | 0.62 (0.58, 0.66) | **<0.001** |
|  | Baseline age |  | -0.61 (-3.64, 2.43) | 0.70 |
|  | Time between SA | 3 months – 1 year | Reference |  |
|  |  | 1-3 years | 5.14 (-36.01, 46.30) | 0.81 |
|  |  | 3-5 years | -55.61 (-128.81, 17.59) | 0.14 |
|  |  | >5 years | -22.18 (-117.14, 72.78) | 0.65 |
| **Sperm concentration** | Baseline sperm concentration |  | 0.75 (0.72, 0.77) | **<0.001** |
|  | Baseline age |  | 0.08 (-0.13, 0.29) | 0.46 |
|  | Time between SA | 3 months – 1 year | Reference |  |
|  |  | 1-3 years | 0.18 (-2.70, 3.06) | 0.90 |
|  |  | 3-5 years | -0.88 (-5.96, 4.20) | 0.73 |
|  |  | >5 years | -5.38 (-11.62, 0.86) | 0.09 |
| **Viability** | Baseline viability |  | 0.01 (0.01, 0.02) | **<0.001** |
|  | Baseline age |  | 0.0001 (-0.007, 0.007) | 0.98 |
|  | Time between SA | 3 months – 1 year | Reference |  |
|  |  | 1-3 years | -0.03 (-0.12, 0.06) | 0.53 |
|  |  | 3-5 years | -0.15 (-0.31, 0.008) | 0.06 |
|  |  | >5 years | 0.05 (-0.17, 0.26) | 0.68 |
| **Normal Morphology** | Baseline normal morphology |  | 0.82 (0.79, 0.85) | <0.001 |
|  | Baseline age |  | -0.06 (-0.10, -0.02) | **0.004** |
|  | Time between SA | 3 months – 1 year | Reference |  |
|  |  | 1-3 years | -0.27 (-0.84, 0.29) | 0.35 |
|  |  | 3-5 years | -1.88 (-2.88, -0.88) | <0.001 |
|  |  | >5 years | -4.55 (-5.92, -3.19) | <0.001 |

Note: CI = confidence interval, SA, semen analysis
