# Supplementary material for: Intra‐individual changes in sperm parameters and total motile count with time among infertile men
Source: Andrology. 2024 Apr 30;13(2):226–33. doi: 10.1111/andr.13638 (PMC11815539; doi:10.1111/andr.13638)
Supplement: Supplementary file 2 — Supporting Information [file ANDR-13-226-s001.docx]

Data presented as median (Q1, Q3). P-values are estimated using the Kruskal-Wallis test.  *Note*: SA, semen analysis; TMC, total motile count

| **Suppl. Table 2.** Change in sperm parameters (last – first SA) per patient in the different age categories according to time between baseline and last semen analyses | | | | | |  |
| --- | --- | --- | --- | --- | --- | --- |
| **P value** | **>5 years** | **3-5 years** | **1-3 years** | **1 year>** | **Parameter** | |
|  |  |  |  |  | **40 years or younger** | |
|  | 63 | 86 | 406 | 787 | **Number of patients** | |
| 0.12 | 0.0 (-0.6, 0.5) | 0.0 (-0.5, 0.5) | 0.0 (-1.0, 0.5) | -0.2 (-1.0, 0.5) | **Volume difference (ml)** | |
| **0.003** | 1.1 (-3.0, 10.0) | 0.8 (-2.6, 11.1) | 0.0 (-7.3, 8.0) | -0.1 (-8.7, 1.1) | **Concentration difference (million/ml)** | |
| **0.001** | 0.7 (-3.6, 6.3) | 0.7 (-5.1, 7.1) | -1.6 (-9.3, 6.3) | -1.8 (-9.3, 2.0) | **Motility difference (%)** | |
| **<0.001** | 0 (0, 5) | 0 (-1, 3) | 0 (-5, 3) | -1.0 (-7.2, 0.0) | **Morphology difference (last-first SA)** | |
| 0.12 | 0 (-5, 10) | 0 (-10, 5) | 0 (-10, 5) | 0 (-10, 5) | **Viability difference (%)** | |
| **0.001** | 0.4 (-1.5, 8.7) | 0.4 (-2.8, 8.2) | -0.1 (-8.9, 4.8) | -0.8 (-6.2, 1.0) | **TMC difference (million)** | |
|  |  |  |  |  | **40-50 years** | |
|  | 9 | 31 | 113 | 254 | **Number of patients** | |
| 0.25 | 0.0 (-0.5, 0.5) | 0.0 (-0.5, 0.5) | 0.0 (-1.0, 0.5) | 0.0 (-1.3, 0.0) | **Volume difference (ml)** | |
| 0.85 | 0.2 (-5.0, 12.8) | 1.0 (-3.0, 9.6) | 0.0 (-9.3, 13.4) | 4.3 (-10.1, 12.7) | **Concentration difference (million/ml)** | |
| 0.09 | 0.0 (-5.5, 7.5) | 1.2 (-4.1, 5.7) | -2.1 (-9.6, 0.5) | 0.0 (-1.3, 4.0) | **Motility difference (%)** | |
| **0.006** | 0 (-1, 1) | 0.0 (-3.0, 3.5) | 0 (-10, 0) | 0.0 (-7.5, 0.0) | **Morphology difference (last-first SA)** | |
| 0.12 | 0.0 (-5.0, 7.5) | 0 (-10, 5) | -5 (-15, 5) | -10.0 (-17.5, -2.5) | **Viability difference (%)** | |
| 0.22 | 0.3 (-3.2, 7.3) | 0.4 (-2.4, 5.0) | -0.7 (-13.7, 4.1) | -0.6 (-4.0, 0.0) | **TMC difference (million)** | |
|  |  |  |  |  | **50 years or older** | |
|  | 0 | 8 | 8 | 47 | **Number of patients** | |
| 0.09 | N/A | 0.1 (-0.2, 0.6) | -0.3 (-0.3, 0.0) | -0.5 (-1.2, -0.1) | **Volume difference (ml)** | |
| 0.21 | N/A | 1.7 (-7.1, 18.5) | -1.3 (-12.8, 1.1) | 9.4 (-2.3, 25.0) | **Concentration difference (million/ml)** | |
| **0.02** | 8 (-6.2, 1.1) | -2.0 (-3.9, 0.4) | 5.5 (2.5, 9.3) | -1.8 (-6.2, 1.1) | **Motility difference (%)** | |
| 0.60 | N/A | 0.0 (-5.0, 2.2) | 0.5 (0.0, 2.5) | 0.0 (-3.8, 0.0) | **Morphology difference (last-first SA)** | |
| 0.80 | N/A | -5 (-15, 5) | -2.5 (-5.0, 0.0) | -2.5 (-5.0, 3.8) | **Viability difference (%)** | |
| 0.79 | N/A | 0.0 (-1.2, 3.7) | 0.5 (-4.3, 1.2) | 0.7 (-0.3, 2.5) | **TMC difference (million)** | |
